# Acute retinal vein occlusion and cystic fibrosis

**DOI:** 10.1186/s40942-018-0129-8

**Published:** 2018-07-18

**Authors:** Matthew R. Starr, Suzanne M. Norby, John P. Scott, Sophie J. Bakri

**Affiliations:** 1 Department of Ophthalmology, Mayo Clinic, 200 First Street Southwest, Rochester, MN 55905 USA; 2 Division of Nephrology and Hypertension, Mayo Clinic, 200 First Street Southwest, Rochester, MN 55905 USA; 3 Department of Pulmonary and Critical Care Medicine, Mayo Clinic, 200 First Street Southwest, Rochester, MN 55905 USA

**Keywords:** Branch retinal vein occlusion, Cystic fibrosis, Fibrinogen, Homocysteine

## Abstract

**Background:**

The ocular manifestations of cystic fibrosis typically present with surface irritation or nyctalopia due to Vitamin A deficiency, however, there have been two previous reports of patients with cystic fibrosis that developed retinal vein occlusions. These reports hypothesized that either elevated fibrinogen levels due to chronic infections or elevated homocysteine levels have predisposed patients with cystic fibrosis to develop retinal vein occlusions.

**Case presentation:**

We present a case of a 35-year-old male with cystic fibrosis complicated by chronic sinusitis with no history of organ transplantation or chronic pulmonary infections who presented with an acute branch retinal vein occlusion in his left eye with associated macular edema. Evaluation revealed an elevated fibrinogen level, while the rest of his workup was relatively unremarkable including a normal homocysteine level. His vision remained 20/20 throughout his care and he did not require treatment of his macular edema.

**Conclusions:**

Patients with cystic fibrosis are at an increased risk of developing retinal vein occlusions likely due to a variety of systemic thrombogenic factors rather than a single risk factor which had been reported previously. Elevated fibrinogen levels in these patients may not be due to chronic infections, but inherent to the cystic fibrosis.

## Background

The ocular manifestations of cystic fibrosis (CF) typically present with ocular surface irritation or nyctalopia due to Vitamin A deficiency secondary to malabsorption; additionally decreased contrast sensitivity, oculosympathetic paresis, decreased lenticular transparency, and optic nerve dysfunction have been reported [1–3]. There have been, however, only two reports of patients with cystic fibrosis with retinal vein occlusions (RVO) [4, 5]. We report a third case of a branch retinal vein occlusion (BRVO) in a young male with CF who was found to have elevated fibrinogen levels on systemic evaluation without an elevation in his homocysteine levels. Previous reports had speculated that each of these markers was the primary risk factor for the development of RVOs in CF patients. It is likely a multifactorial risk, though, given the other significant underlying thrombogenic risk factors in CF patients.

## Case presentation

**Fig. 1:**
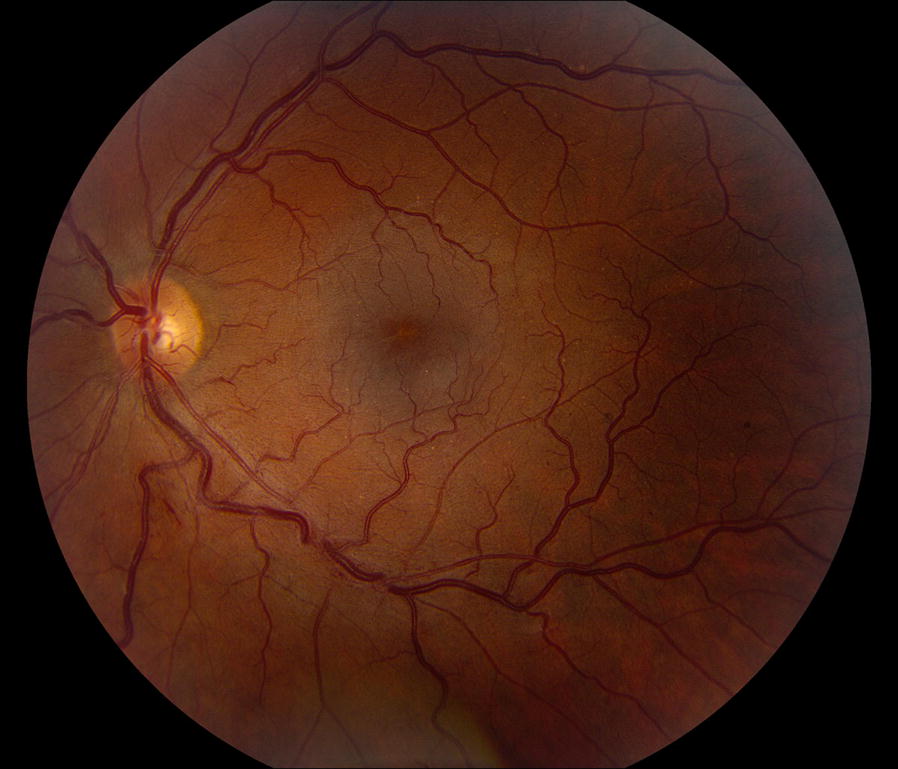
Color fundus photograph of the left eye of a 35 year-old male with cystic fibrosis and inferior branch retinal vein occlusion. Notable flame hemorrhages along the inferior nerve fiber layer with marked arteriovenous nicking and minimal macular edema. Arteriovenous nicking is present along both the superior and inferior arcades which corresponds to the patient’s known systemic hypertension

**Fig. 2:**
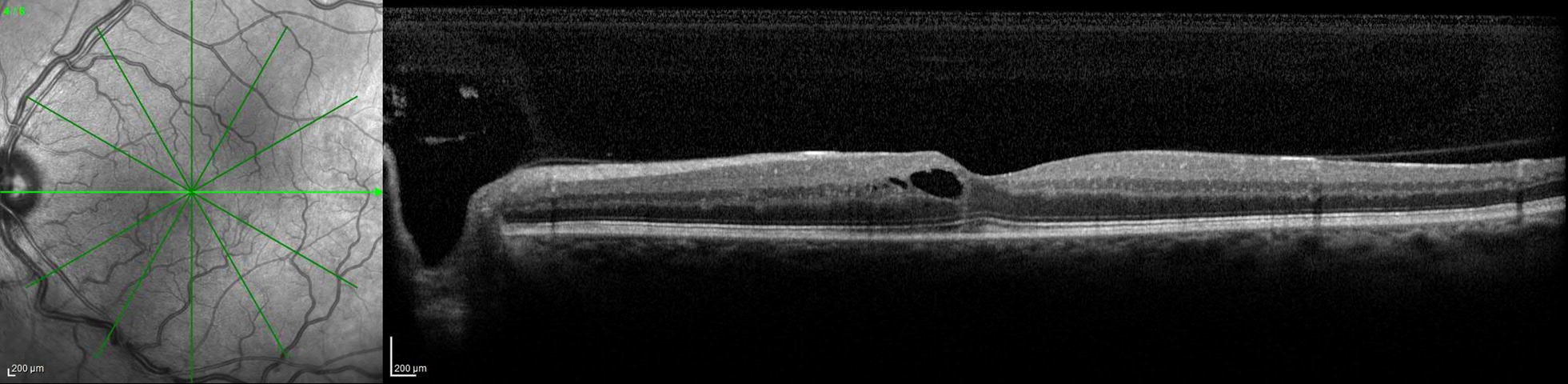
Optical coherence tomography of the left eye with intraretinal cystic fluid near the fovea. There is trace subfoveal fluid with a small amount of hyper-reflective material directly beneath and inferior to the fovea

**Fig. 3:**
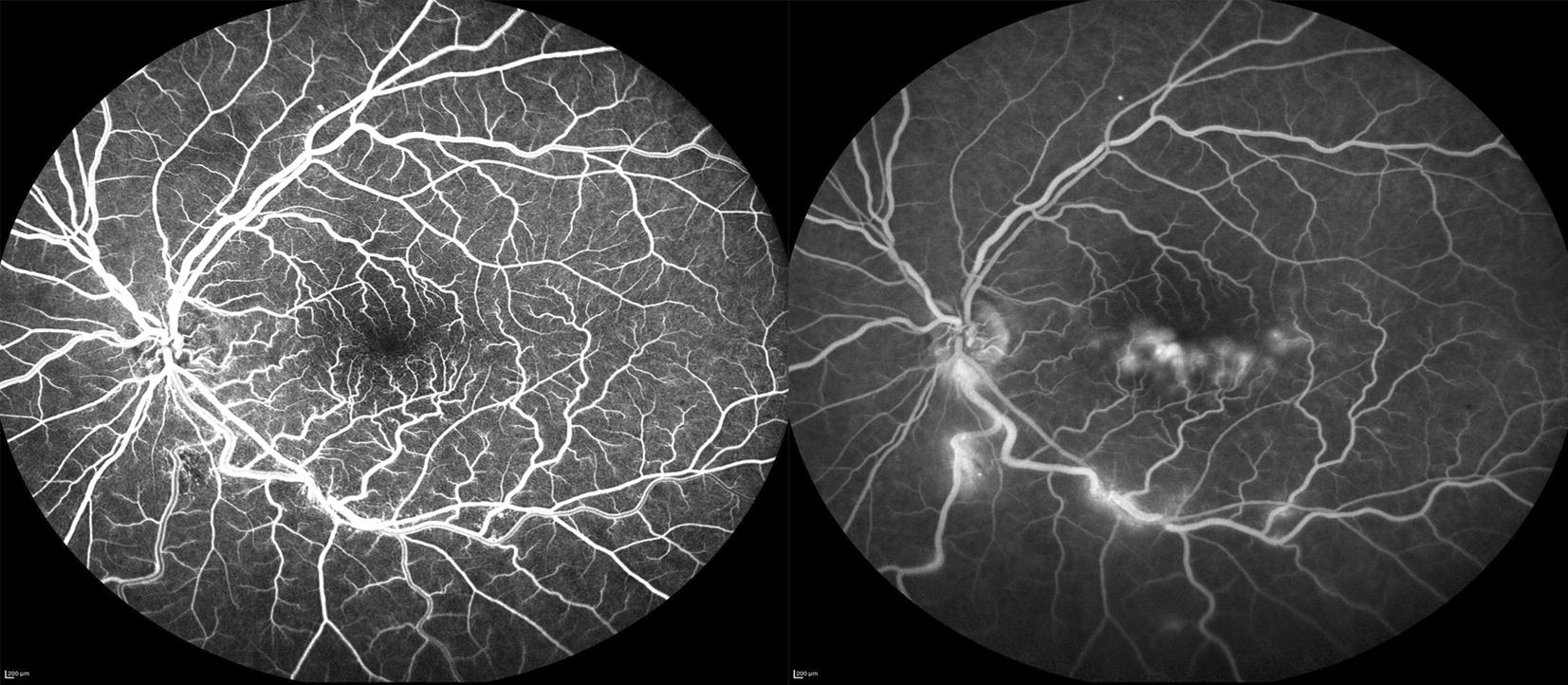
Early (left) and late (right) fluorescein angiogram (FA) of the left eye with delayed venous filling inferiorly during the early peak phase with a small area of blocking inferior to the disc corresponding to hemorrhage on exam (left). Telangiectatic vessels are noted in the early phase FA as well. In the late phase FA, there is leakage inferiorly along the arcade at areas of AV nicking. Also seen during the late phase FA is leakage at the inferior edge of the fovea corresponding to the intraretinal fluid seen on OCT (right)

A 35-year-old male presented with constant blurry vision in his left eye for 6 weeks. His past medical history was significant for CF complicated by chronic pancreatic insufficiency leading to insulin dependent diabetes mellitus, chronic sinusitis, hypertension, iron deficiency anemia, and obstructive sleep apnea. His only previous surgery was a combined sinus surgery consisting of bilateral maxillary antrostomies, ethmoidectomies, sphenoidectomies, and frontal sinusotomies performed 7 months prior to presentation. Medications at the time of presentation included albuterol, azithromycin, itraconazole, insulin, lisinopril, pancreatic enzymes, and sulfamethoxazole-trimethoprim. He denied any tobacco or drug use. His visual acuity at presentation was 20/20 in both eyes, intraocular pressure was 18 OD and 16 OS, and his pupils were 4 mm and reactive in both eyes. His anterior exam was unremarkable, but his posterior exam in the left eye was significant for intraretinal hemorrhages along the nerve fiber layer with associated retinal thickening in the inferior macula consistent with a BRVO (Fig. 1). Optical coherence tomography revealed mild intraretinal and trace subfoveal fluid in the left eye (Fig. 2). The findings were confirmed with fluorescein angiogram which revealed delayed venous filling in the inferior venous arcade (Fig. 3).

**Fig. 4:**
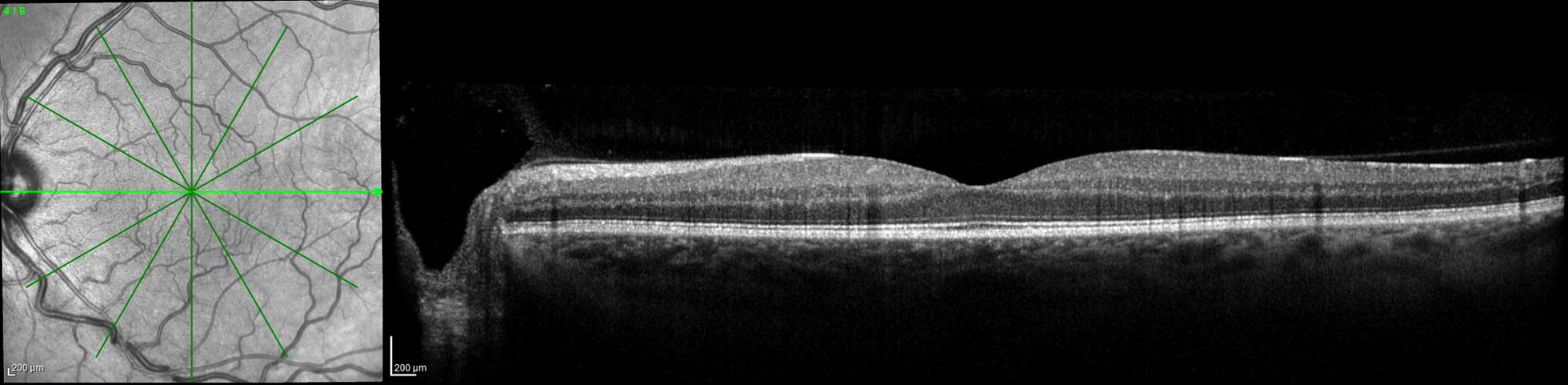
Optical coherence tomography of the left eye 6 months after initial presentation with resolution of the intraretinal and subretinal near the fovea. The foveal contour and photoreceptor layers remain intact

His blood pressure readings had been consistently between 110 and 145 systolic over 70–85 diastolic in the past year, and his most recent hemoglobin A1c was 6.9% 4 months prior to presentation. Laboratory workup revealed normal complete blood cell count, Vitamin A and D levels, prothrombin time, thrombin time, antithrombin activity, Protein C and S activity, cardiolipins, dilute Russell’s viper venom time, homocysteine level, and a negative prothrombin G20210A mutation. A complete metabolic panel was notable for a mild elevation in glucose of 121 mg/dL (70–100 mg/dL). Antinuclear antibody was weakly elevated at 2.1 units (≤ 1 unit), and sedimentation rate was 24 mm/h (0–22 mm/h) with normal C-reactive protein. The only significantly abnormal lab was a fibrinogen level of 479 mg/dL (200–375 mg/dL).The patient was observed without treatment given his visual acuity was 20/20 and the macular fluid did not involve the fovea (Fig. 1). Six months after presentation he remained 20/20 in both eyes and the macular fluid had resolved (Fig. 4) with careful observation.

## Discussion and conclusions

In the first report by Gelman and colleagues, they describe a 31-year-old male who developed a central retinal vein occlusion (CRVO) in one eye and then 4 years later a sequential CRVO in the contralateral eye. An initial limited workup after the first CRVO was unrevealing, but when the patient developed a second CRVO, the workup was repeated with additional testing that included homocysteine and gamma globulin levels, both of which were elevated. The second CRVO developed 9 months after the initiation of intravenous immunoglobulin therapy for his cystic fibrosis and was felt to contribute to the hypergammaglobulinemia. They concluded that the elevated homocysteine level in this patient was likely due to deficiencies in the methionine-homocysteine cycle, a trait which can be seen in patients with CF [6]. Homocysteine is a well-known risk factor for the development of retinal vein occlusions [7, 8], and these authors felt that both the elevated homocysteine level combined with the hypergammaglobulinemia synergistically led to the development of sequential CRVOs in this patient.

The second case report described by Hiscox et al. was of a 35-year-old male with an asymptomatic branch retinal vein occlusion (BRVO) in his left eye discovered on funduscopic exam. Limited workup revealed an elevated fibrinogen level, but a homocysteine level was not reported for this patient. It was felt that this patient’s chronic pulmonary infections led to a persistent pro-inflammatory state leading to elevations in fibrinogen which played a role in the development of the BRVO.

It is possible that the elevated fibrinogen level in our patient was associated with his chronic sinusitis, as fibrinogen is an acute phase reactant. Being an acute phase reactant, elevated fibrinogen levels can also be seen following surgery, with an infection, or with certain medications [9]. Our patient’s most recent surgery was 7 months prior to presentation, he did not have an elevated white blood cell count indicating an infection, and none of his medications are known to be associated with alterations in fibrinogen levels. Recently, though, a study by Rottner and colleagues showed that patients with CF may intrinsically develop a pro-inflammatory state regardless of their chronic infections [10]. These authors postulated that the mutation in the cystic fibrosis transmembrane conductance regulator in CF patients leads to activation of pro-inflammatory cellular pathways. This inflammation leads to elevated fibrinogen levels and other inflammatory markers in patients with CF [11]. Elevated fibrinogen, specifically, confers an increased risk of retinal vascular abnormalities, including CRVO and BRVO [12]. Regardless of the fibrinogen levels though, all three patients had systemic vascular risk factors likely leading to the retinal venous occlusions in each patient, despite aggressive medical management. Our patient notably had obstructive sleep apnea, insulin dependent diabetes mellitus, and hypertension, all prominent vascular risk factors that can lead to vein occlusions.

It is also worth noting that our patient did not have elevated homocysteine levels as seen in the first patient with sequential CRVOs. Hyperhomocysteinemia is certainly an important risk factor for vein occlusions and is likely a significant risk factor for all patients with CF given the increased risk these patients have of developing hyperhomocysteinemia [6]. However, it is possible that RVOs in patients with CF are multifactorial and not due to a single risk factor. Patients with CF are predisposed to developing pro-thrombotic conditions such as diabetes, hypertension, and hyperlipidemia as well as elevated fibrinogen, hyperhomocysteinemia, and hypergammaglobulinemia. The combination of all of these factors predisposes CF patients to developing RVOs. No definitive conclusions can be made from a single case report, but it is important for ophthalmologists to recognize CF as a possible risk factor for the development of retinal vascular abnormalities even though these patients are typically much younger than the average patient with a retinal artery or vein occlusion.

## Abbreviations

CF: cystic fibrosis
RVO: retinal vein occlusion
BRVO: branch retinal vein occlusion
CRVO: central retinal vein occlusion
